# An Ultrasound-Based Liquid Pressure Measurement Method in Small Diameter Pipelines Considering the Installation and Temperature

**DOI:** 10.3390/s150408253

**Published:** 2015-04-09

**Authors:** Xue Li, Zhengxiang Song

**Affiliations:** State Key Laboratory of Electrical Insulation and Power Equipment, Xi’an Jiaotong University, Xi’an 710049, China; E-Mail: zxsong@mail.xjtu.edu.cn

**Keywords:** liquid pressure measurement, ultrasonic transducer, installation difference compensation, temperature compensation

## Abstract

Liquid pressure is a key parameter for detecting and judging faults in hydraulic mechanisms, but traditional measurement methods have many deficiencies. An effective non-intrusive method using an ultrasound-based technique to measure liquid pressure in small diameter (less than 15 mm) pipelines is presented in this paper. The proposed method is based on the principle that the transmission speed of an ultrasonic wave in a Kneser liquid correlates with liquid pressure. Liquid pressure was calculated using the variation of ultrasonic propagation time in a liquid under different pressures: 0 Pa and X Pa. In this research the time difference was obtained by an electrical processing approach and was accurately measured to the nanosecond level through a high-resolution time measurement module. Because installation differences and liquid temperatures could influence the measurement accuracy, a special type of circuit called automatic gain control (AGC) circuit and a new back propagation network (BPN) model accounting for liquid temperature were employed to improve the measurement results. The corresponding pressure values were finally obtained by utilizing the relationship between time difference, transient temperature and liquid pressure. An experimental pressure measurement platform was built and the experimental results confirm that the proposed method has good measurement accuracy.

## 1. Introduction

Hydraulic systems are widely used in various fields, such as water transportation, port construction, the power industry, urban construction, oil and gas transportation, *etc*. Liquid pressure is one of the basic parameters in any hydraulic system, so the measurement of liquid pressure plays an important role in the monitoring the condition of and fault diagnosis of hydraulic systems.

Conventional liquid pressure measurement methods are intrusive, requiring drilling into the pipelines to install a pressure gauge or pressure sensor [1]. This could affect both the sealing performance of the hydraulic system and the load characteristics of the pipeline. Several authors have proposed non-intrusive measurement methods based on elastic deformation of the pipeline [2,3]. A small elastic deformation of the pipe’s external diameter occurs due to the liquid pressure inside, so the liquid pressure can be measured by establishing a relationship between them. However, in most cases, the elastic deformation of the pipe is so small that it is difficult to measure accurately.

In recent years, ultrasonic non-destructive testing methods have been widely used in flow meters [4,5], stress testing [6], viscosity measurements [7], density measurements [8] and distance measurements [9] because of their fast response, strong penetration, powerful anti-jamming features and harmlessness. The advantages of ultrasonic technology have also become apparent in the measurement of liquid pressure.

Until now, many researches on the measurement of pipe pressure utilizing ultrasonic waves have been conducted [10,11,12,13]. One of them was based on the non-linear acoustical interaction between a high frequency ultrasonic beam and the low frequency flow fluctuation caused by circulation pumps. An ultrasonic probe which was installed on one side of pipe emitted an ultrasonic beam, then the beam crossed the pipe wall and was collected by a receiving probe on the opposite side. Through phase modulation of the ultrasonic signal, a pressure value could be calculated by using the relationship between pressure and phase [10]. However, in small diameter pipes, wave superposition will occur because ultrasound travels quickly, which leads to measurement difficulties. Another measurement strategy was proposed based on the relationship between the velocity of the acoustic signal traveling through the liquid and pipe pressure. Ultrasonic probes were mounted on both sides of the pipe wall. The acoustic velocity was reflected by measuring the time difference between two ultrasonic waves using a cross-correlation [11]. However, this method had poor real-time performance and a narrow application range because of its complicated algorithm. Besides, the transmission characteristics of ultrasound are easily affected by liquid temperature and installation differences, which makes these measurement methods inaccurate and even erroneous.

Therefore, the objective of this research was to accurately measure liquid pressure in small diameter pipelines by applying a non-intrusive ultrasonic technique compensating for the influence of installation differences and liquid temperature. In this paper, a new measurement method directly based on time differences is proposed. Shear wave transducers were used to eliminate the wave superposition in pipes with inner diameter of less than 15 mm. In addition, a kind of circuit called AGC circuit and a BP network algorithm were employed to improve the measurement accuracy.

## 2. Measurement Principle

### 2.1. Theoretical Background

Ultrasound is an acoustic wave with a frequency ranging from 20 kHz to 0.5 GHz. Its propagation velocity in a liquid is related to liquid pressure. In different liquids, there are different mathematical relationships between the ultrasonic propagation velocity and liquid pressure. In acoustics hydraulic oil belongs to the Kneser liquid class. In a Kneser fluid, the velocity of an ultrasonic wave would increase evenly with the increase of liquid pressure at a certain temperature [14]. A liquid pressure measurement method in a hydraulic system filled with Kneser liquid is discussed in this article.

Piezoelectric probes for generating and detecting ultrasonic waves have widespread application. The generation of ultrasonic waves is achieved by means of piezoelectricity. Ultrasonic waves can also be converted into electrical signals based on reverse piezoelectricity. For this reason the same probe can be used as a transmitter as well as a receiver [15,16,17].

**Figure 1:**
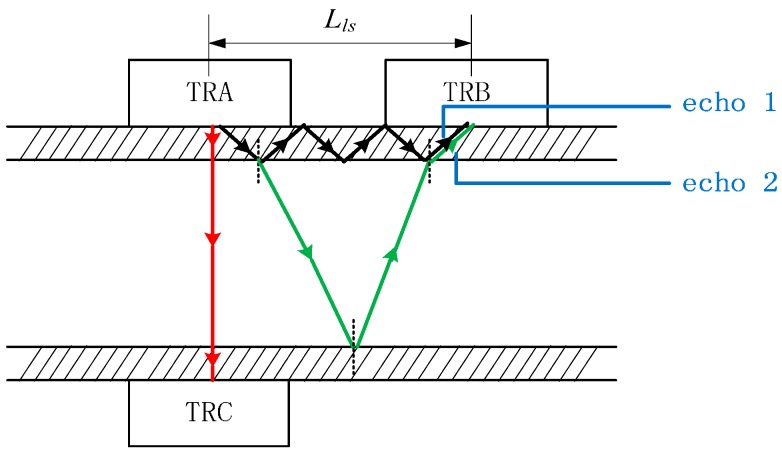
Schematic diagram of the ultrasonic propagation path.

Two shear wave transducers, defined as TRA and TRB, are mounted as shown in Figure 1, and the propagation path of the ultrasound signal is also shown. First, a shear wave generated by TRA transmits into the pipe wall. Then, a partial wave is reflected, and continues to spread inside the pipe wall until finally received by TRB. The partial wave is refracted and converted to a longitudinal wave at the solid-liquid junction because an ultrasonic shear wave can only spread in a solid while a longitudinal ultrasonic wave can spread in liquids and solids. After that, the longitudinal wave is reflected at the liquid-solid junction and continues to spread in the liquid. Finally, it is refracted into the TRB, so the echo signals received by TRB mainly contain the echo 1 which only propagates in the pipe wall and echo 2 which propagates in the liquid and pipe wall. TRB can also be used as a transmitting probe, and TRA as a receiving probe to repeat the same process. Compared with TRA and TRC which serve as longitudinal wave transducers as shown in Figure 1, the propagation distance is much longer. Thus, it is helpful to avoid wave superposition in pipelines with small diameter.

### 2.2. Measurement Principle

As mentioned above, the ultrasonic velocity in oil correlates with the liquid pressure. Since the variation of ultrasonic velocity cannot be measured directly, it can be represented by the variation of propagation time. As Figure 1 shows, the time delay from emission of the ultrasound to reception of echo 2 is a combination of the propagation time of the longitudinal wave in the fluid and the propagation time of the shear wave inside the tube wall (*t'*). Besides, there is an acoustic and electrical time delay *t''*, which includes the unexpected delays of probes, coupling materials, connection cables, receiving circuitry *etc*. *t'* and *t''* remain almost unchanged while the liquid pressure changes. Assume the transmission distance of an acoustic wave in the liquid is *L*, which does not change with liquid pressure. Then the time delay *t_1_* taken from emission of ultrasound to reception of echo 2 when pressure is not exerted on the fluid is:

(1) $$t_{1}=\frac{L}{v_{0}}+t^{\prime }+t^{\prime \prime }$$

here, *v_0_* is the propagation velocity of an ultrasonic wave in a fluid with no pressure.

The time delay *t_2_* with X Pa pressure is:

(2) $$t_{2}=\frac{L}{v_{0}+\Delta v}+t^{\prime }+t^{\prime \prime }$$

here, ∆*v* is the variation of the ultrasonic propagation velocity after pressure is exerted on the fluid.

Thus, time difference ∆*t* between these two time delays is:

(3) $$\Delta t=t_{1}-t_{2}=\frac{L}{v_{0}}+t^{\prime }+t^{\prime \prime }-(\frac{L}{v_{0}+\Delta v}+t^{\prime }+t^{\prime \prime })=\frac{L\cdot \Delta v}{v_{0}(v_{0}+\Delta v)}$$

Therefore, the influence of the pipe’s thickness and *t''* can be effectively eliminated. Since *L* and *v_0_* will be difficult to get, the liquid pressure is directly obtained by the relationship between ∆*t* and *P* in this paper. This means that there is no need to calculate the variation of ultrasonic velocity via time difference. The proposed measurement method is based on the principle described above.

### 2.3. The Effect and Improvement of Installation Difference

**Figure 2:**
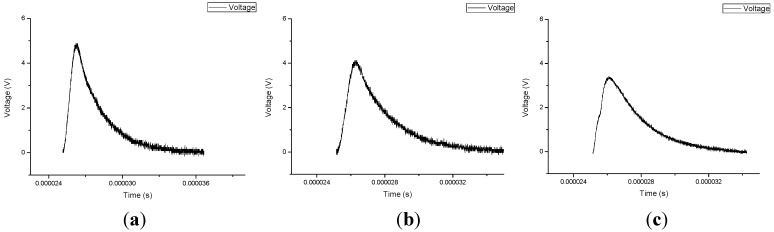
Echo signals with different installation conditions.

The probes are repeatedly installed on the same pipe, and the difference between each installation condition is defined as the installation difference. This includes differences in slack level, degree of parallelism and the contact situation between the probes and the pipeline. Under a certain pressure, there are different echo signals which are reflected by the pipe wall after the waves transmit in the liquid (echo 2) caused by the influence of the installation differences. This difference is mainly reflected in the intensity of echo signals, as illustrated in Figure 2. It will finally develop into two results: an echo signal that is too weak to be detected, in which case the probes should be reinstalled or a diversity of echo signals appear, which will lead to the fluctuation of the time difference under the same pressure. This has a serious impact on measurement accuracy. Accordingly, a special circuit called an automatic gain control (AGC) circuit was introduced to improve the measurements.

The AGC circuit is one of the most important circuits in electronic equipment, especially in communication receivers and radar receivers. It is used to maintain the output level in a certain range when the input signal changes within a large range [18]. Due to its better control and faster response, a digital closed-loop AGC system is employed in this paper.

**Figure 3:**
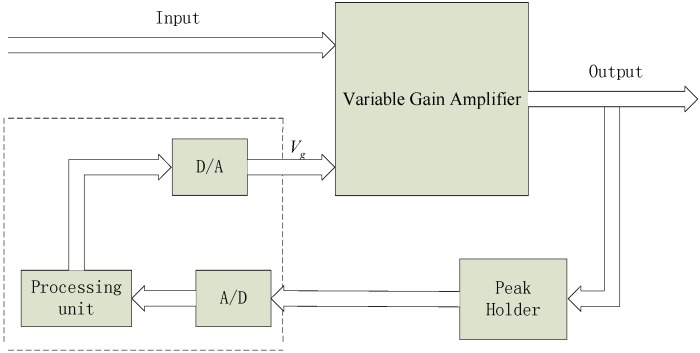
Block diagram of the AGC system.

As seen in Figure 3, an AGC system was designed, which contained a peak holder, a circuit generating a gain control voltage and a variable gain amplifier. Above all, the amplitude was detected and maintained with the utilization of the peak holder. Then, an A/D converter was used to sample the peak voltage and a D/A converter was applied to export the gain control voltage calculated by the processing unit. Finally, the variable gain amplifier was controlled to adjust the amplification by the gain voltage, which made output signal almost constant.

The algorithm in processing unit is when the peak voltage is smaller than the lower limit, print out error to notify users to reinstall; when the peak voltage is within the normal range, calculate and output the gain control voltage *V_g_* based on Equation (4):

(4) $$V_{g}=(V_{d}/V_{p})\times k$$

where *V_d_* is the desired voltage set by the users; *V_p_* is the peak voltage of the output signal; *k* is the ratio coefficient, whose units are volts.

### 2.4. Temperature Compensation

In a Kneser liquid, the velocity of an ultrasonic wave will decrease evenly as the liquid temperature increases under a certain pressure. Experiments show that the rate of change of the acoustic velocity caused by temperature is 0.2% per degree Celsius [14]. Moreover, due to the impact of the work environment, system characteristics, mechanical operation or other factors, the liquid temperature is always changing. Therefore, it is necessary to compensate for the influence of temperature in order to realize accurate liquid pressure measurements.

Existing compensation methods are mostly static, which compensate the influence of temperature via a simple mathematical formula. The value calculated by the proposed formula is subtracted from the measurement result. In order to acquire higher accuracy results, a real-time temperature compensation method is proposed in this paper. Specifically, the variation of ultrasonic propagation velocity can be seen as a function of liquid pressure and temperature. According to Equation (3), the following formula can be written:

(5) $$\Delta t=\frac{L\cdot f(\text{P,}T)}{v_{0}(v_{0}+f(\text{P,}T))}$$

Thus, P could be expressed as the function of *△t* and T:

(6) P=g(Δt,T)

Function g is nonlinear and it cannot be expressed precisely through a mathematical expression. Thus, the neural network analysis method which has remarkable nonlinear fitting capability is applied in this article to simulate this relationship.

**Figure 4:**
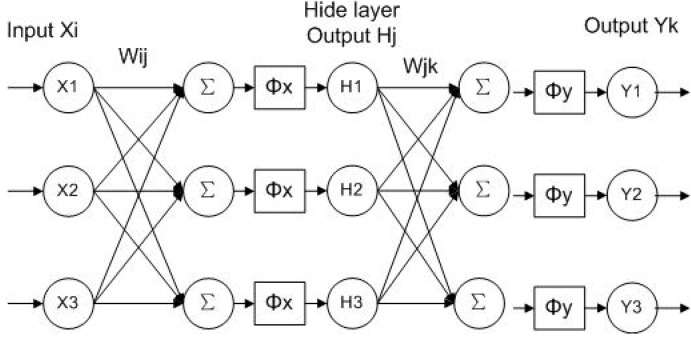
Structure of three-layer BP neural network.

A type of neural network, called back propagation network (BPN), is adopted, because of its simple structure, adjustable parameters, and more training algorithms [19]. According to the Kolmogorov theorem, a three-layer BP network can exactly approach any nonlinear function. The typical layer structure of a BPN, which is composed of an input layer, a hidden layer and an output layer, is displayed in Figure 4.

The learning procedure of a BP neural network is as follows: first, the input layer acts on the output layer through the hidden layer; then the error between the actual out vector and the desired output vector is calculated and back propagated; after that, each connection weight in the network is revised so as to minimize the error. The procedure is repeated until the error reaches the set value. At this point, learning stops and network is generated.

In this paper, a relationship among time difference ∆*t*, temperature *T* and pressure *P* is established by a BP neural algorithm. Therefore, after inputting the time difference and temperature into the trained neural network, the liquid pressure in the pipeline can be acquired from the output of the network.

## 3. Experimental Set-up and Method

**Figure 5:**
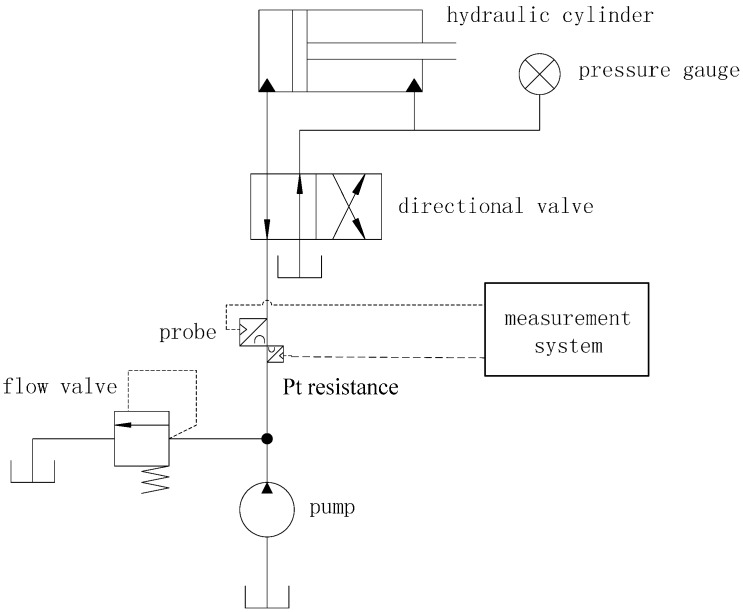
Schematic diagram of the experimental platform.

An experiment platform, illustrated in Figure 5, was built to verify the validity of the proposed method. It consisted of a hydraulic system and a measurement system. A hand pump (model number: SB-12.5, Tonghua Hydraulic Factory, Yancheng, China) was employed to produce pressure in the pipe. A digital pressure gauge (model number: CW-SY-60, Xian Chuangwei Measurement and Control Instrument Co., Ltd., Xi’an, China) with an error of no more than 0.01 MPa, was utilized to meter the desired pressure value. The type of oil is 10# aircraft hydraulic oil. The material of the pipe is steel, and the inner diameter of the pipe is 12 mm. In order to acquire better transmission and echo signals, a 2.5P8*12k1.5 type of probe (Changan Testing Company, Xi’an, China) was employed. The lateral separation between TRA and TRB (*L_ls_* in Figure 1) is fixed. Besides, lubricating grease, which is a very good ultrasonic coupling medium, was used as the coupling medium between the probes and the pipe wall.

**Figure 6:**
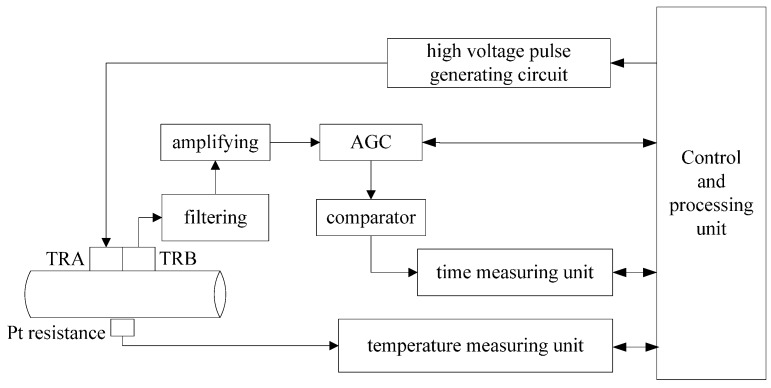
Block diagram of the measuring system.

The measurement system was designed to measure the liquid pressure in the hydraulic system based on the proposed method. Figure 6 shows its implementation with TRA serving as transmitter and TRB serving as receiver. Firstly, by applying a high spike voltage pulse to the piezoelectric probe TRA, an ultrasound wave with the frequency 2.5 MHz was generated. The amplitude of the spike was −500 V and the pulse width of the spike was about 0.4 μs. After transmitting in the pipe wall and the hydraulic oil, the ultrasonic echo was received by TRB.

**Figure 7:**
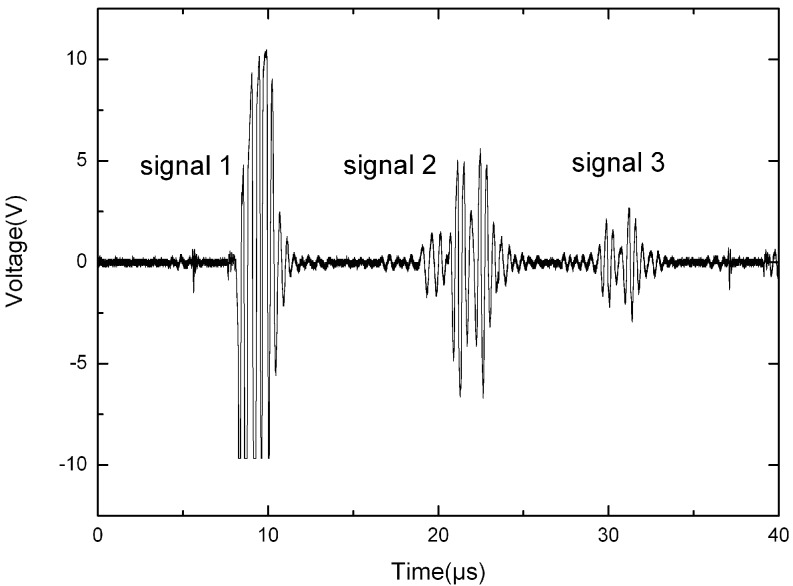
Waveform at the receiving probe.

The ultrasonic waveform shown in Figure 7 was the processing result of the initial signal received by TRB. Signal 1 was the induction signal of the high voltage pulse. Signals 2 and 3 were the electrical signals produced by the acoustic signals echoes 1 and 2 reaching TRB separately.

**Figure 8:**
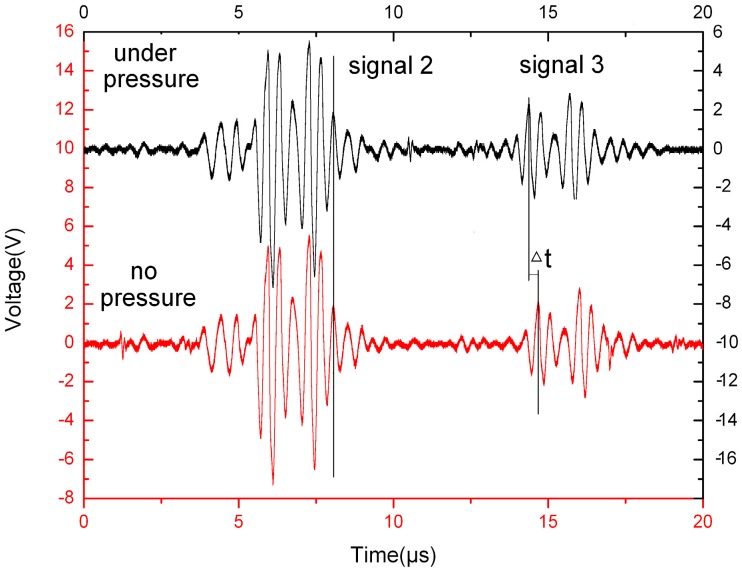
The comparison of ultrasonic echoes with or without pressure.

Figure 8 illustrates the waveforms of the magnified echoes with pressure or not. After X Pa pressure was applied to the liquid, there was no motion in the time-axis for signal 2 while signal 3 moved significantly in the time-axis. The reason for this phenomenon is that liquid pressure could only change the ultrasonic velocity in the liquid, but has no influence on the ultrasonic velocity in the pipe wall.

Secondly, the echo signal was processed as follows: it was amplified and filtered in the first place; then signal 3 was taken out by a selector and chosen as the input of the AGC circuit; the output signal of the AGC circuit was send to a comparator, and was finally converted to a square ware signal.

Thirdly, the square wave signal was sent to the time measuring unit, where a Time-to-Digital Converter (TDC) chip (ACAM, Stutensee-Blankenloch, Germany) was employed to meter the time delay from the emission of ultrasound to the reception of signal 3. The TDC chips use internal propagation delays of signals through gates to measure time intervals with very high precision. They are actuated by a START signal and stopped by a STOP signal. In this measurement module, signal 1 was the START signal and signal 3 was the STOP signal. Finally, measurement results were sent back to the processing unit for further calculations.

In the meantime, a Pt resistance (Pt 500) clung to the pipe wall was employed to measure the transient liquid temperature. Because the pipes in the hydraulic systems are sealed, the temperature of the liquid inside the pipe cannot be measured directly. Besides, the temperature in the working site changes slowly. And the diameter of the pipe is small. Therefore, the measurement results can reflect the temperature of the liquid inside the pipe. Results acquired by the temperature measurement unit were also sent back to the processing system for further analysis. TRA and TRB were used as the ultrasonic transmitting and receiving probe separately during each measurement process. As a result, two sets of data have been collected. The average value of these two sets, including ∆*t_A_* and ∆*t_B_*, was used as final result ∆*t* in order to reduce the error caused by the different device parameters.

## 4. Results and Discussion

### 4.1. Installation Difference

In order to study the influence of installation differences and verify the effectiveness of the AGC circuit, experiments with different installation conditions have been done when the applied liquid pressure is 6.7 MPa. In the different installation conditions, the probes and pipeline were in different relative positions. For comparison, results with or without the AGC processing circuit are displayed in Table 1. In the system without AGC circuit, the echo signal was processed as follows: after amplification and filtering, the echo signal was turned into an envelope signal by a detection circuit; then it was converted to a square wave signal through a comparator circuit. Other parts were the same as the measurement system mentioned before.

**Table 1:** Experimental results with different installation conditions.

Installation Conditions	∆*t/ns*	
	With AGC	Without AGC
1	285	298
2	283	288
3	286	300
4	280	286
5	281	300

It can be seen from Table 1 the variation of the time difference in the measurement system with AGC circuit is much smaller than that in the measurement system without the AGC circuit. This means that the installation difference has an obvious influence on the measurement results and this influence could be mitigated by the AGC circuit.

### 4.2. Temperature

As mentioned previously, temperature has an impact on the velocity of ultrasonic wave in liquid, which makes the measurement of liquid pressure imprecise. Therefore, the following experiment was designed to study the influence of this factor. In the experiment, a heater was used to raise the liquid temperature, which was measured in real time by the Pt resistance mounted on the pipe wall. The results are shown in Table 2.

**Table 2:** Experimental results with different pressures and temperatures.

P(MPa)	T(°C)	∆*t* (ns)
0.00	21.7	0.0
0.00	33.3	713.5
0.00	31.8	461.5
7.00	31.2	101.5
6.00	30.1	95.5
5.00	28.8	82.5
4.00	27.6	64.0
3.18	26.6	53.0
3.00	26.4	50.5
2.28	25.6	43.0
2.00	25.3	40.0
0.00	25.1	122.5
7.00	24.2	−208.5
6.00	23.6	−191.0
4.95	23.0	−173.0
4.31	22.6	−158.5
3.02	21.8	−135.0

Therein, the zero point was defined as the point where the temperature was 21.7 °C, and the pressure was 0 MPa. *△t* represents the average time difference between the results with different pressure or temperature and the zero value. As for the tested oil, the ultrasonic propagation time in it will increase with the increase of liquid temperature, but decrease with the increase of liquid pressure. Therefore, under the influence of these two opposing factors, the average time difference *△t* may be positive or negative. Since the temperature rise is small, there is an assumption that the thermal expansion of the pipe is negligible.

**Figure 9:**
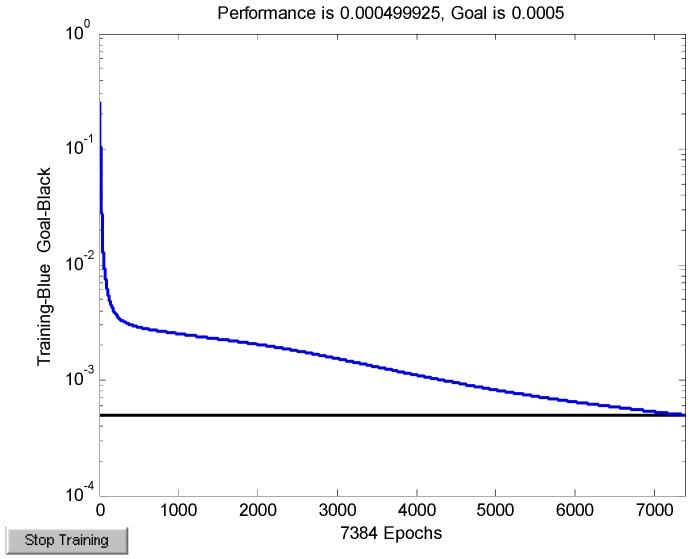
The training error in the neural network.

The BPN model mentioned in Section 2.4 has been built in Matlab. Temperature *T* and time difference *△t* were defined as the input of the BPN model, and liquid pressure *P* in the pipeline was the output. Determining the number of hidden nodes is really a problem because it could affect the generalization ability, fault-tolerant performance and training time of the network. In this paper, the hidden nodes are set to 8 after repeated testing and good fitting results could be achieved. The Tan-Sigmoid function and Log-sigmoid function were chosen to be activation functions of the hidden layer and the output layer, respectively. The square error was set as 0.0005. The training error in the neural network is shown in Figure 9.

Another set of data which was obtained by the same experimental approach was used to test the trained network. The data and processing results are shown in Table 3. The desired pressure is the result measured by the digital pressure gauge, and the actual pressure is the result calculated by the trained network. It can be seen that the relative error between the actual output and the desired output is less than 6%, which meets the demands of an engineering application. In the proposed method, ACAM’s TDC chip with a resolution in the picosecond range was employed to meter the time difference. So the time difference between 0 Pa and 0.1 MPa, which is about 4 ns, can be measured by the high-resolution time measurement module. That means the sensitivity of the measurement method is 0.1 MPa. The uncertainty of the method is mainly caused by installation differences and inconsistent ultrasound output.

**Table 3:** Testing results with the neural network.

T (°C)	∆*t* (ns)	Desired Pressure (MPa)	Actual Pressure (MPa)	Relative Error (%)
21.9	0.0	0.00	0.1177	-
31.6	592.5	0.00	0.0008	-
29.4	67.0	6.10	6.4336	5.5
28.5	62.0	5.00	5.2696	5.4
24.5	98.5	0.00	0.0904	-
24.3	−222.5	7.00	6.8130	−2.7
23.6	−211.5	6.00	6.2365	3.9
23.0	−198.5	5.00	5.2575	5.2
22.5	−182.0	4.08	4.2300	3.7
22.1	−172.0	3.35	3.4348	2.5

## 5. Conclusions

This study focused on the measurement of liquid pressure in small diameter pipelines. As the liquid pressure in the pipe changes, the transmission speed of an ultrasonic wave in the liquid will also change. By measuring the difference in propagation time of an ultrasonic wave in oil with and without pressure, the pressure value could be calculated. Owing to the small variance of the time difference in pipelines with small diameter, shear wave transducers were employed and a high-precision time measuring system was designed. Two factors, including installation differences and liquid temperature, will influence the measurement accuracy. In this paper, an AGC system is designed to reduce the impact of installation differences by maintaining an almost constant output. A new BPN model which uses both time difference and liquid temperature measured in real time as inputs is built to ameliorate the influence of liquid temperature. The construction of the measurement system and the analysis of the experimental data are shown in this article. The experimental results indicate that the proposed method is effective and accurate in the measurement of liquid pressure in small diameter pipes.

Although the accuracy of the proposed method is lower than that of the traditional pressure gage, it has the advantages of non-intrusive measurement. In addition, the accuracy of the proposed method is as good as the accuracy of the non-intrusive methods described in the Introduction. However, compared with these non-intrusive methods, the proposed method has better real-time performance and wider application range. It can mitigate the impact of installation difference and liquid temperature effectively and measure the liquid pressure in small diameter pipes correctly.
